# Lactate-Based Model Predictive Control Strategy of Cell Growth for Cell Therapy Applications

**DOI:** 10.3390/bioengineering7030078

**Published:** 2020-07-20

**Authors:** Kathleen Van Beylen, Ali Youssef, Alberto Peña Fernández, Toon Lambrechts, Ioannis Papantoniou, Jean-Marie Aerts

**Affiliations:** 1 Department of Biosystems, Division Animal and Human Health Engineering, M3-BIORES: Measure, Model & Manage Bioresponses Laboratory, KU Leuven, Kasteelpark Arenberg 30, 3001 Heverlee, Belgium; kathleen.vanbeylen@kuleuven.be (K.V.B.); ali.youssef@kuleuven.be (A.Y.); alberto.penafernandez@kuleuven.be (A.P.F.); toon.lambrechts@mycellhub.com (T.L.); 2 Prometheus, Division of Skeletal Tissue Engineering, KU Leuven, Onderwijs en Navorsing 1, Herestraat 49, 3000 Leuven, Belgium; ioannis.papantoniou@kuleuven.be; 3 Skeletal Biology and Engineering Research Centre, Onderwijs en Navorsing 1, Herestraat 49, 3000 Leuven, Belgium; 4 Institute of Chemical Engineering Sciences, Foundation for Research and Technology—Hellas (FORTH), 26504 Patras, Greece

**Keywords:** model predictive control, bio-process, cell growth, lactate, advanced therapy medicinal products

## Abstract

Implementing a personalised feeding strategy for each individual batch of a bioprocess could significantly reduce the unnecessary costs of overfeeding the cells. This paper uses lactate measurements during the cell culture process as an indication of cell growth to adapt the feeding strategy accordingly. For this purpose, a model predictive control is used to follow this a priori determined reference trajectory of cumulative lactate. Human progenitor cells from three different donors, which were cultivated in 12-well plates for five days using six different feeding strategies, are used as references. Each experimental set-up is performed in triplicate and for each run an individualised model-based predictive control (MPC) controller is developed. All process models exhibit an accuracy of 99.80% ± 0.02%, and all simulations to reproduce each experimental run, using the data as a reference trajectory, reached their target with a 98.64% ± 0.10% accuracy on average. This work represents a promising framework to control the cell growth through adapting the feeding strategy based on lactate measurements.

## 1. Introduction

Cell-based products receiving market approval are increasing over the last years. The European Medicine Agency (EMA) has approved 14 medicinal products based on gene therapies, cell therapies or tissue engineering, also called advanced therapies for the European market [1]. The U.S. Food and Drug Administration (FDA) has approved 17 cellular or gene therapy products [2]. Compared to other pharmaceuticals such as small molecule drugs or biologics, the active pharmaceutical ingredient (API) of these cell-based therapies is living cells. An example of such a cell-based therapy is chimeric antigen receptor (CAR) T-cell therapy, where the patient is injected with human immune cells, which are modified to target cancer cells [3]. Another type of cell-based therapy is skeletal tissue engineering, where a cell-based implant is used to regenerate cartilage or bone in the patient instead of using a prosthetic implant, which has the disadvantage that it will need to be replaced within 10–15 years [4]. Besides being the active component of the final medicinal product, cells can also be used as a tool in the manufacturing process to produce the final product, such as extracellular vesicles [5].

With the introduction of this promising group of cell-based or cell-derived products, the necessity to transform the emerging cell therapy and regenerative medicine industrial sector towards a BioPharma 4.0 sector is growing. This revolution should build on a strong IT infrastructure combined with automation technologies in order to use continuous data to steer and optimise bioprocesses in real-time without the need for human interaction [6]. Closely monitoring and controlling the bioprocess tackles the challenge of irreproducible manufacturing processes that are often seen for (personalised) cell-based therapies. This bioprocess variability is inherent to donor variability, the time-varying characteristics of progenitor cells (such as phenotype) and the complexity of living systems [7].

Progenitor cell expansion is a crucial process step whereby clinically relevant numbers are produced typically ranging between 5 × 10^7^–10^8^ [8,9]. Currently, progenitor cell expansion relies on fixed protocols which do not take into account the particularities of the cell type, donor characteristics or the batch, leading to suboptimal outcomes [10]. In order to reduce this variability, the process has to remain within predefined boundaries during the whole production process, which is possible by actively adapting critical process parameters (CPP) during the cell expansion process, based on the characteristics and individual needs of a batch. The retuning of the process parameters should be done in a way that would enable the process to follow a predefined (reference) trajectory, providing optimal conditions for the cultured cells. Due to the inherent variability of cells and the time-varying dynamics of the process, modelling and controlling the cell growth is challenging [11].

Active control of cell culture bioprocesses will also result in lower batch-to-batch variability. Without any monitoring or control of cell culture, there could be a high amount of batch rejections due to results of in-process or finished product testing falling out of the predefined boundaries of the validated process. These specifications are described in quality control documents approved by health authorities and are set to assure product quality and safety. The amount of “out of specifications” test results of two different commercial cell therapies was recently described in the biologics license application (BLA) submission of Kymriah^®^ and Yescarta^®^. Novartis reported 7% and 9% manufacturing failures for Kymriah batches, whereas Kite reported 1% for Yescarta batches [12]. Novartis disclosed that all out of specifications (OOS) results were caused by viability problems, resulting in final products with a viability lower than 80%. The challenge lies in the nature of cell products having an inherent variability and complexity.

Therefore, in this work, a model-based predictive control (MPC) system is proposed as a potential solution to the aforementioned challenges of inherent variability and time-varying dynamics of the cell culture process [13]. MPC exhibits several interesting features, such as intuitive concepts, easy tuning and the ability to control a range of simple and complex phenomena, including systems with time delays, non-minimum phase dynamics, dead times, multivariable cases and instability [14]. While dealing with all these challenges, the MPC can easily incorporate constraints and tailor formulated control objectives [12,13]. Model predictive control offers several important advantages: (1) the process model captures the dynamic and static interactions between input, output and disturbance variables; (2) constraints on inputs and outputs are considered in a systematic manner in the cost function and (3) accurate model predictions can provide early warnings of potential problems [13].

Several studies have investigated the benefits of controlling the environment of cell culture vessels such as dissolved oxygen tension (dO_2_), temperature and CO_2_ [15]. Instead of using these standard physicochemical process parameters to control the bioprocess, this paper will develop a method to control the metabolic responses of the cells. This metabolic response is measured off-line and is used as an indication of the cell growth, which can only be measured at the end of the bioprocess of adherent cells. An interesting metabolic response to use as an indirect measure for cell growth in a high glucose medium is the cumulative lactate production of the cells over the culture period. Using lactate measures has the advantage, in an environment with excess amount of glucose, that the ratio between lactate production and glucose consumption is a known value (two) based on the anaerobic glycolysis pathway [16]. In high glucose environments, measurements of glucose have a low sensitivity compared to lactate. Lactate concentrations are low in fresh medium and are produced by the cells, resulting in higher sensitivity and indication of whether or not cells are alive. Another advantage is controlling the pH, since this is related to the lactate concentration [17,18]. The control of this pH is important because an increase in extracellular acidosis, i.e., a value below 6.7, leads to a higher amount of apoptosis [19,20].

Furthermore, lowering the lactate concentration by replacing the media for 100%, 50% or 0% of the total working volume has been reported to have a significant effect on the cell growth [15].

The aim of this paper is to describe a framework for controlling process parameters of the cell expansion process based on lactate measurements in combination with a model predictive control approach. As a proof of concept we used lactate measures, but depending on the considered application, the input and output could be chosen differently, taking into account specific process parameters and quality attributes. For example, in low glucose environments, it would be interesting to change the measurement to glucose. By controlling the process parameters, the cell growth can be directed towards a predefined reference trajectory. This research demonstrated the intended goal using experimental data in combination with control strategy simulations.

## 2. Materials and Methods

### 2.1. Cell Culture Experiments

In order to develop this framework, we performed experiments on human periosteum-derived cells (hPDCs) and studied their metabolic responses during their cell expansion process. Cell proliferation was the aimed output. This cell growth was represented here by the cumulative lactate produced by the cells. As an input to control the cell growth, we investigated the effect of the total amount of replaced medium.

#### 2.1.1. Cell Culture

The hPDCs used in this study were obtained from periosteal biopsies with patients’ informed consent. The performed biopsy procedures, as described by [21], were approved by the Ethics Committee for Human Medical Research (KU Leuven). These cells were expanded until passage 4 and frozen. Culture medium consisted of high glucose Dulbecco’s modified Eagle’s medium (DMEM + GlutaMAX^TM^ + pyruvate, Gibco^TM^ by Thermo Fisher Scientific, Waltham, MA, USA), supplemented with 10% (v/v) heparin-free pooled human platelet lysate (Stemulate^TM^ by Cook Regentec, Indianapolis, IN, USA) and 1% antibiotic-antimycotic (Gibco^TM^ by Thermo Fisher Scientific).

The cell culture experiment started by thawing three frozen vials, each containing 1 million hPDC cells from a different donor. The cells from these three donors were seeded in three different T175 flask at passage 5 with 27 mL culture medium and incubated in a humidified atmosphere of 90% at 37 °C and 5% CO_2_. The culture medium used during the experiment was DMEM supplemented with only 7.5% hPL instead of 10%, which was used for general cell culture expansion and storage. The reason for lowering the amount of hPL is based on knowledge from previous experiments, indicating cells cultured in 7.5% hPL as the condition with the lowest medium cost per population doubling (data not included). Cells were subjected to a 100% medium replacement on day 2 and harvested on day 4 with TrypLE (Gibco^TM^ by Thermo Fisher Scientific). This passaging was repeated once again, with the same seeding density of 5700 cells∙cm^−2^.

#### 2.1.2. Experimental Set-Up

Cells were harvested after the second expansion step and seeded into 6 different 12-well plates (72 wells), each well with a density of 3300 cells∙cm^−2^ in 1 mL of DMEM medium supplemented with 7.5% hPL. Reducing the seeding density from the previous 5700 cells∙cm^−2^, which was used for expanding and storing of cells, to 3300 cells∙cm^−2^ was, on the one hand, based on previous experiments. These experiments indicated a seeding density of 3300 cells∙cm^−2^ to be a more cost-effective use of the culture vessel, due to a lower population doubling time and similar cell number harvested at the end of the cell culture. On the other hand, a lower seeding density would also provide more cell culture time before reaching 80% of confluency, resulting in a higher amount of input and output data points. The cells were cultured during 5 days while the medium was replaced according to 6 different medium replacement strategies, as indicated in Table 1.

All conditions were performed for three different donors in triplicates (54 wells). In addition a control condition was set up in each of the six 12-well plates in triplicates (18 wells), which had the same medium replacement scheme as condition 6, but the cells were from a pool of the three different donors to account for possible well plate differences.

#### 2.1.3. Lactate Measurements and Cell Counts

During the 5 days of cell culture, 100 µL medium samples were taken every day from each and stored at −80 °C. Therefore, a minimum of 10% medium replacement was required. The medium samples were analysed for lactate with the CEDEX medium analyser (Roche, Custom Biotech, Belgium) after thawing. After five days of cell culture expansion, the cells were harvested using TrypLE express and counted with trypan blue 0.25% using a Bürker haemocytometer.

### 2.2. Model-Based Control and Optimisation

#### 2.2.1. System Identification and Modelling

**Figure 1:**
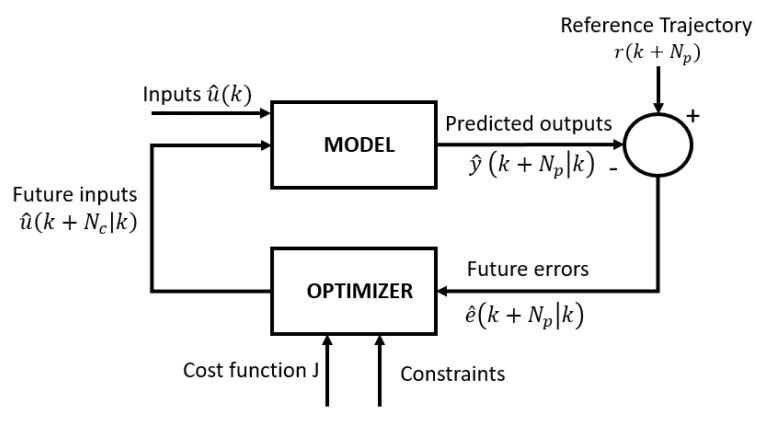
Model predictive controller scheme [22].

The main goal of this work is to (1) optimise the cell proliferation, combined with (2) minimising the use of medium, which can be achieved by tuning a process parameter to steer the process towards a defined growth trajectory. In order to solve this optimisation problem, a model-based predictive control (MPC) approach is used, which is shown in Figure 1.

The control strategy consists of a dynamic model to forecast the future behaviour of the system (predicted outputs $\hat{y}(k+N_{p}|k)$, at time k with prediction horizon $N_{p}$). This predictive knowledge is used in combination with the past knowledge of previous input and output measurements of the system and a reference trajectory ($r\left(k+N_{p}\right)$) to calculate the future errors ($\hat{e}(k+N_{p}|k)$). The optimiser will take these errors in to account, together with the cost function (J) and the constraints, to formulate the optimal control decision (future inputs $\hat{u}\left(k+N_{c}|k\right)$, estimated at time k with control horizon $N_{c}$) to be used as inputs to minimise the deviation from the reference trajectory [23].

A first step in developing a model-based controller is to develop a model of the process. When no readily available mechanistic model or knowledge is available, a model can be identified based on measuring process inputs and outputs. Several methods can be used, but an approach that has been proven successful in many applications is system identification. This approach assumes that the observed input–output relations of the system are the manifestation of the dominant processes occurring within the system under study. Typically, a transfer function (TF) model structure is estimated as an objective and the parsimonious mathematical description of the process is considered [24].

The reason for using a data-based model predictive controller is based on the multiple advantages it has regarding controlling and optimising systems compared to classical proportional–integral–derivative (PID) controllers [25]. The model will predict the lactate increase and use time varying parameters combined with an a priori defined reference trajectory required for the complex and time-varying nature of the cells. Furthermore, the model is able to include feedback knowledge of experiments and extract the main processes to see the effect on the growth. In addition, it can take into account constraints on the input and output variables, use short prediction horizons and avoid time delay problems.

#### 2.2.2. Interpolated Data

One of the challenges faced during the present study was the sparsity of the data points, with only one data point every 24 h. Therefore, an interpolation step was needed, for which the method of piecewise linear interpolation was used. In order to do this, all collected data points are used and the data in between are estimated using a linear function [26]. For a dataset of *n* points ($t_{1},y_{1})$, .., ($t_{n},y_{n})$ with $t_{1}<t_{n}$, the piecewise linear interpolation for point t situated at $t_{k}<t<t_{k+1}$, is described by

(1) $$y\left(t\right)=y_{k}+\frac{y_{k+1}-y_{k}}{t_{k+1}-t_{k}}\cdot \left(t-t_{k}\right),$$

where *y* (mmol) is again the accumulated lactate produced and *t* (days) is the culture period in days. The values ($t_{k}$, $y_{k}$) and ($t_{k+1}$, $y_{k+1}$) are collected data points, whereas (t,y(t)) is an interpolated data point. The resulted interpolated data were used as a reference trajectory in the simulation step for the developed model predictive controller.

#### 2.2.3. Prediction Model

The MPC approach requires a dynamic model which forecasts the output, in this case the cell growth. Furthermore, the model relates the process parameters, used as inputs, to this desired output. The goal of this work is to estimate the growth of the cells during the cell culturing phase. However, since adherent cells cannot be measured directly in this phase, an indirect measure of cell growth is used, namely, accumulated lactate produced by the cells during proliferation.

The advantage of the previous mentioned system-identification methods, such as transfer function models, is that they develop the process models directly based on measured process data and thus can take into account differences between cell types and/or time-varying characteristics.

**Figure 2:**
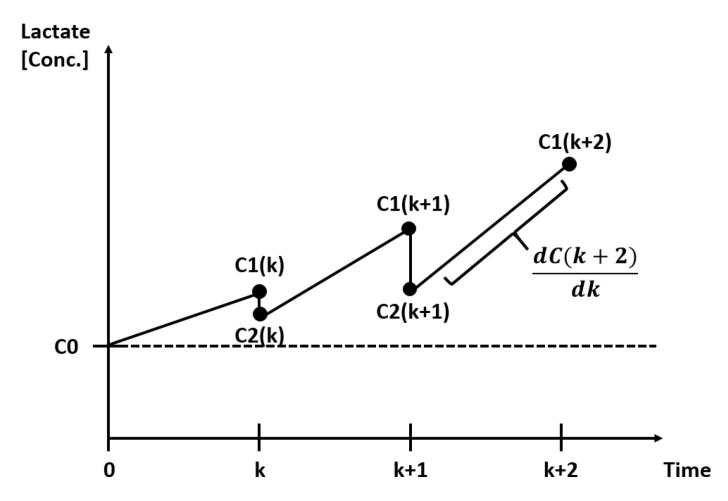
Simplified lactate concentration graph during a cell expansion period with medium replacements at time points k, k+1 *and* k+2.

Figure 2 shows a representation of the lactate concentrations over time, with medium replacements at certain time points *k*.

At time zero, the cell culture has an initial lactate concentration which is equal to the concentration in fresh medium and is called the baseline concentration $C_{0}$. While the cells proliferate, they consume nutrients such as glucose and produce waste products such as lactate. Therefore, the lactate concentration increases between time zero and *k* from the initial $C_{0}$ until $C_{1}\left(k\right)$. At time *k*, the medium is replaced with U(k) as a percentage of the working volume of the vessel. After medium replacement, the lactate concentration $C_{1}\left(k\right)$ decreases to $C_{2}\left(k\right)$ as described in the following equation:

(2) $$C_{2}\left(k\right)=C_{1}\left(k\right)-C_{1}\left(k\right)U\left(k\right)+C_{0}U\left(k\right).$$

To control this lactate production, the amount of medium used to replenish the cells can be used as the manipulated process parameter (or control input).

A data-based mechanistic model approach was used to describe the effect of changing the medium replacement on the cumulative lactate production. A transfer function input–output model structure is used for system model identification, as little knowledge of the complex cell behaviour is required a priori.

In this research, dynamic auto-regressive exogenous (DARX) variables are estimated using the CAPTAIN toolbox [27] in MATLAB version 2018b. The DARX model is used in the analysis to allow a changing relation between medium replacement and accumulated lactate during the cell culture period [28]. The model structure is described as follows [22,23]:

(3) $$y_{t}=\frac{B\left(z^{-1},t\right)}{A\left(z^{-1},t\right)}u_{t-\delta }+\frac{1}{A\left(z^{-1},t\right)}e_{t},$$

where $y_{t}$ is the output (accumulated lactate (mmol)) of the system and $u_{t-\delta }$ the input (accumulated medium replaced (mL) with a certain time delay δ. The additive noise $e_{t}$ is assumed to have a zero mean and uncorrelated variance $N\left(0,\sigma^{2}\right)$. The series A and B have time varying parameters described by the following equations:

(4) $$A\left(z^{-1},t\right)=1+a_{1,t}z^{-1}+a_{2,t}z^{-2}+\cdots +a_{n,t}z^{-n_{a}}$$

(5) $$B\left(z^{-1},t\right)=b_{0,t}+b_{1,t}z^{-1}+b_{2,t}z^{-2}+\cdots +b_{m,t}z^{-n_{b}},$$

where the backward shift operator $z^{-1}$, applied on the model parameters $a_{i,t}$ and $b_{i,t}$, can also be expressed as:

(6) $$a_{i,t}z^{-i}=a_{i,t}\left(t-i\right).$$

To obtain the relation between input and output, estimated by the polynomials A and B, experiments were performed. These experiments changed the process parameter (u, medium replacement) while measuring the effect on the output (y, cumulative lactate concentration). The model parameters were estimated using refined instrumental variable (RIV) algorithms [27]. The most suitable reduced order model structure was selected based on two identification criteria, namely, the coefficient of determination $R^{2}$ and Young identification criterion (YIC). The orders of these polynomials in Equations (4) and (5) are $n_{a}$ and $n_{b}$. For these data, model orders between 1 and 2 for *n* and *m* respectively were evaluated, including time delays between 0 and 1. The best fit was obtained using first order polynomials with a fixed $a_{1}$ parameter in time and a variable $b_{0}$ during all the different time points. The accuracy of this fit is measured with MATLAB version 2018b using normalised root mean square error (NRMSE) with the goodness of fit function. This method is described as follows:

(7) $$\mathrm{NRMSE}=1-\frac{\left\|y_{ref}-y_{fit}\right\|}{\left\|y_{ref}-mean\left(y_{ref}\right)\right\|}$$

where $y_{fit}$, the modelled data, compared to $y_{ref}$, the reference data. The NRMSE equals 1 for a perfect fit.

#### 2.2.4. Cost Function

The optimal process parameter values are those which steer the system towards the reference trajectory function. These values are calculated as the ones minimising a controller’s cost function. This cost function consists of one term to minimise the difference between the predicted output ($\hat{y}$) and the reference trajectory (r), and another term to minimise the change of the control signal (Δu) (i.e., the replaced medium volume). This equation is as follows:

(8) $$J\left(Nc,Np\right)=\sum_{j=1}^{Np}\delta \left(j\right){\left[\hat{y}\left(k+j|k\right)-r\left(k+j\right)\right]}^{2}+\sum_{j=1}^{Nc}\lambda \left(j\right){\left[\Delta u\left(k+j-1\right)\right]}^{2},$$

where Nc is the control horizon, Np is the prediction horizon (time points where *y* is controlled to follow *r*) and δ,λ are used as weights to create a relevance ranking [22,29].

#### 2.2.5. Constraints

The solutions to optimise the system are subject to constraints. The input, manipulated to control the system, could be restricted by physical boundaries. For example, replacing the medium for 100% in certain vessels is impossible without the risk of removing cells together with the medium. In addition, the output of the system could also be restricted to assure product quality, feasibility or safety. For example, the lactate concentration of the cell culture system is limited to avoid toxic lactate levels, meaning a value of 20 mM [30]. There was no need to implement these constraints in the current work, since the toxic lactate threshold was never reached in these experiments, not even for the condition of minimal lactate replacement.

#### 2.2.6. Simulation

In this paper, the use of a model-based predictive control approach was evaluated for cell growth control by quantifying the performance of the controller based on simulated control actions. More specifically, the experimental data were used to identify time-varying transfer function models describing the dynamic relations between cumulative lactate concentrations and medium refreshments and these models were used in combination with the control algorithms to simulate the needed medium refreshments. The reference trajectory for cumulative lactate concentration was assumed to be the cumulative lactate concentrations actually measured for each condition.

## 3. Results

### 3.1. Collected Data

**Figure 3:**
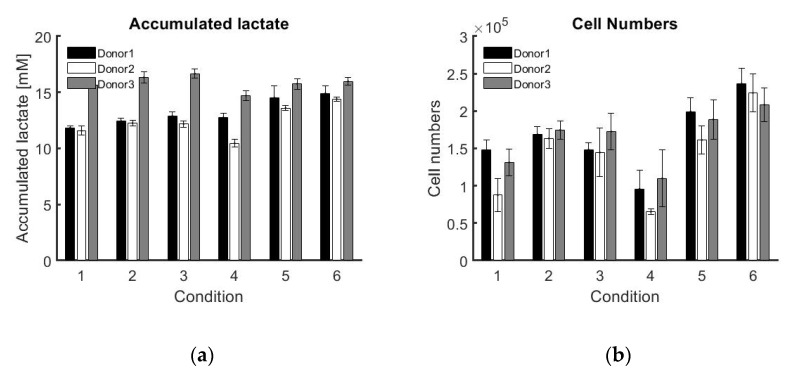
Average results with error bars for the triplicate experiments of each individual donor with each specific medium replacement strategy. (**a**) Total accumulated lactate produced after 5 days of cell culture (mM). (**b**) Cell numbers counted at the end of the cell culture period.

Figure 3 shows the amount of accumulated lactate produced and the cell number after the five days of cell expansion. These results are summarised in Table 2, and show the average of the triplicates for the different donors and different medium replacement conditions, which were explained in Table 1.

**Figure 4:**
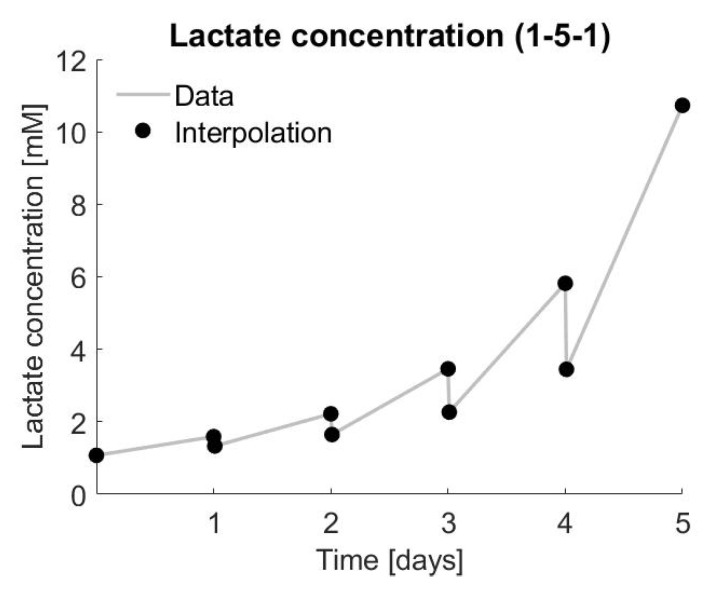
Lactate concentration over time (days) for the results of donor 1, condition 5 and triplicate 1.

Figure 4 represents an example of measured lactate concentration over time, similar to Figure 2, but adapted to the data from the experiments with donor 1, condition 5 and triplicate 1.

Table 3 represents how efficiently the amount of medium is used for proliferation by the cells. This is calculated by dividing the total amount of cells by the total amount of medium supplied during the cell expansion. The table indicates that the most efficient medium replacement strategy, meaning the most cells per amount of medium used, is donor- and not method-dependent. All three different donors require different medium replacement strategies. However, giving the cells the highest amount of medium (condition 6 with 100% medium replacement every 24 h) always results in the highest amount of cells at the end of the expansion. Also, the lowest amount of medium replacement always results in the lowest amount of cells at the end of the expansion.

Therefore, to develop a model predictive controller, it is always necessary to keep in mind what the goal or reference is. If the goal is to predict the feeding strategy of the cells in order to reach the highest amount of cells, the controller would suggest to replace the medium as much as possible. The downside is that resources are wasted due to unnecessary medium replacements. A more interesting question would be to ask the controller how much medium should be replaced to reach, for example, 80% of the total amount of cells according to a maximum medium replacement strategy (condition 6) in the same amount of time. Or another question could be, in a case where a patient has a procedure scheduled in fixed amount of time, e.g., eight weeks: how much medium should be provided to the cells to reach the therapeutically-required amount of cells in eight weeks?

Table 4 represents the results of the average amount of lactate produced by each cell at the end of the cell expansion. This relation is interesting for translating the accumulated amount of lactate produced to the amount of cells. However, this number differs for each donor and differs even more between different medium-replacement strategies. Condition 6, in which the medium is replaced 100% every day, could be a representation of how the cells produce lactate in an optimal environment. Condition 4, in which only 10% of the medium is replaced every day, has a significantly higher amount of lactate produced over the expansion period, which is due to either a lack of nutrients and growth factors, or inhibiting factors such as lactate itself.

One of the biological reasons for this difference in lactate produced by cells could be that cells die due to this lactate inhibition or nutrient and growth factor depletion. Therefore, less cells are counted in the end than actually lived, causing a higher lactate∙cell^−1^ ratio [31]. Another reason could be that cells are changing their metabolic profiles [32].

### 3.2. Interpolated Data

When using the piecewise linear interpolation method, the fit was 100%, since all data points are being used. The piecewise linear interpolation methods were further used to interpolate the sparse data set. Instead of using only one data point every day, the data are interpolated to one data point every hour, which reflects a more realistic approach for field conditions.

### 3.3. Prediction Model

**Figure 5:**
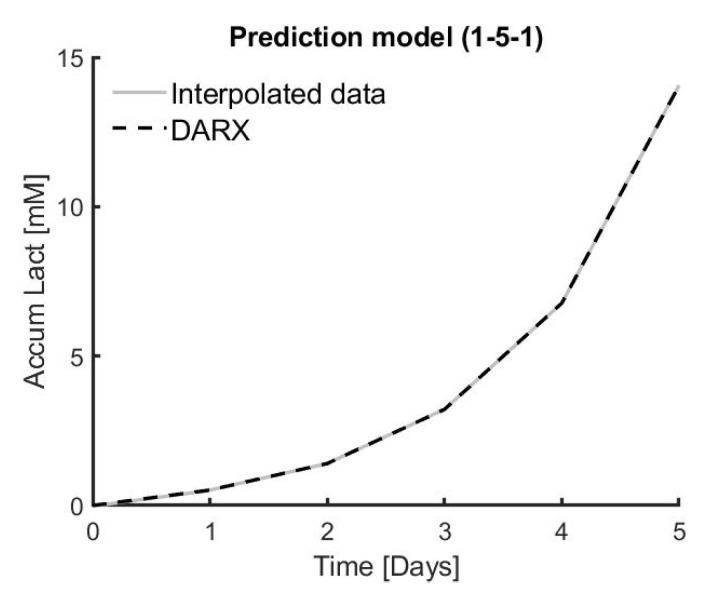
Visualisation of dynamic auto-regressive exogenous (DARX) model for accumalated lactate (mM) compared to the interpolated data for the experiment of donor 1, condition 5 and triplicate 1.

The model parameters for the DARX model, represented in Equation (3), were obtained using first order polynomials with a fixed $a_{1}$ parameter (cf. Equation (4)) in time and a variable $b_{0}$ (cf. Equation (5)) during all the different time points. The accuracy of this DARX model compared to the piecewise interpolated output is shown in Table 5 and visualised in Figure 5.

**Figure 6:**
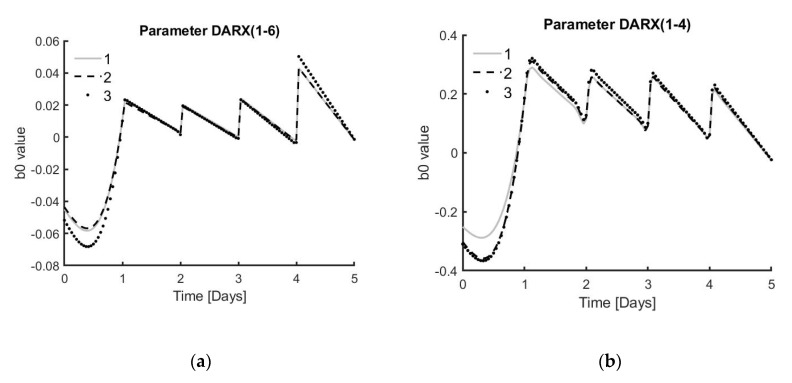
Graphs exhibiting the *b_0,t_* values over time. (**a**) *b_0,t_* values for triplicates of donor 1 under condition 6, which means 100% medium replacement every 24 h. (**b**) *b_0,t_* values for triplicates of donor 1 under condition 4, which means only 10% medium replacement every 24 h.

Using a fixed parameter $a_{1}$ and a dynamic parameter *b_0,t_* results in only one parameter adjusting to the dynamics of the system, making the interpretation of the changes easier. From Figure 6 it can be deducted that *b_0,t_* is an indicator of how much the cells are competing for the medium. On one hand, if the parameter exhibits an overall low absolute value, as seen in Figure 6a, it indicates that the cells have leftover medium that is not used and will be replaced unnecessarily, which means that resources are wasted. On the other hand, if an overall higher absolute value is attained, as seen in Figure 6b, then the cells do not have enough medium to fulfil their potential growth.

### 3.4. Simulation of the Model Predictive Controller

**Figure 7:**
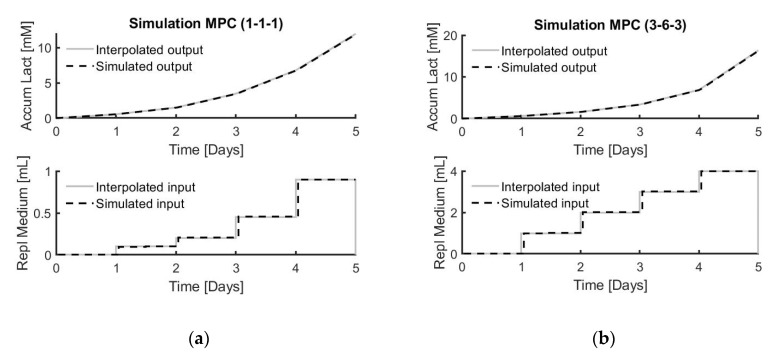
Top graph shows the interpolated output (accumulated lactate (mM)) compared to the simulated output. Bottom graph represents the interpolated input (total replaced medium (mL)) compared to the simulated input. (**a**) model-based predictive control (MPC) simulation applied to donor 1, condition 1 and triplicate 1; (**b**) MPC simulation applied to donor 3, condition 6 and triplicate 3.

MPC simulations were performed based on the identified prediction models for each type of medium replacement strategy. An example for condition 1 and condition 6 are given in Figure 7, with accumulated lactate produced by the cells as target output and accumulated amount of medium replacement as an input variable.

The goodness of fit between the controller’s input suggestions and output and the experimental data are summarised in Table 6 and Table 7.

## 4. Discussion

Monitoring and controlling the cell growth is crucial when developing a large-scale reproducible cell culture process. However, there are currently no standardised methods to sample the amount of cells during a cell culture expansion in tissue flasks or hollow fibre bioreactors. Previous studies have therefore investigated the benefits of controlling the environment of the cell culture vessels using standard physicochemical process parameters [15]. In addition, other studies developed potential soft sensors using the metabolic responses of the cells to control the process, mostly glucose concentration [33,34]. This work used this metabolic soft sensor concept by measuring the lactate concentration off-line and used it as an indication of the cell growth, which can otherwise only be measured at the end of the bioprocess.

Choosing the correct control strategy for this framework results in high accuracy between the experimental data and the simulated data. Many different control strategies have been explored in fermentation processes [35], some for mammalian cells [15,17], and a few for human cells [36]. These control strategies are built on either user experience, a process model or historical data [35]. Each strategy has its own benefits and disadvantages. Using an approach based only on user experience has the advantage that it can be quickly applied to a new system without the need for historical data or a process model. However, these approaches, such as probing control [37] or fuzzy control [38], are running behind the action, because they act when the current state is not ideal, without an optimal strategy for the whole process. When there is a large amount of historical data, interesting approaches are artificial neural networks [20,21] or statistical process controls [39]. However, for cell therapy bioprocesses this is mostly not the case, since these data are very process-specific and cannot be extrapolated for different cell types, batch sizes or in autologous applications, which are donor-specific. Mechanistic mathematical approaches encounter the same difficulty, because their specific sets of kinetic parameters have to be redefined for each specific process, requiring many specific data sets. A mathematical model, for example, one that describes the exponential growth of cells in combination with consumption nutrients and production waste products [36], is useful for the prediction of an average control strategy for that cell type. However, the downside of these mathematical models is that they contain cell-lineage-specific kinetics parameters from literature and should be updated for every stage of that cell lineage, e.g., proliferation or differentiation [40].

In cases where there is a process model available, the preferred choice would be to use model-based predictive control (MPC), because it can deal with non-linear dynamics, unpredictable disturbances and provides insight for the user [35]. Other attempts at controlling bioprocesses using an MPC have been made. One of them consisted of controlling the glucose concentration to maintain more than a certain threshold of 11 mM in a 15 L fed-batch system [34]. To achieve this, they used a non-linear model-based predictive control to adapt the feed rate based on a mechanistic mathematical model which describes the cell growth and metabolism. However, the main problem was the process–model mismatch, which is inherent to the variability of a bioprocess. They also compared an off-line measurement method with 12 h between samples to an on-line spectroscopy technique sampling every six minutes. The problem with on-line glucose methods was a high sample-to-noise ratio. Another study tried to avoid this problem of the high cost and noise of on-line glucose sensors by developing a soft sensor [33]. This soft sensor uses cumulative oxygen transfer rates, calculated using several on-line measured variables. It defines the correlation between the on-line soft sensor and the real glucose concentration by comparing off-line measures of glucose every 24 h to recalculate the correlation.

What was still missing from most current control strategies is the combination of a model predictive control with an adaptive control strategy to avoid the process–model mismatch [34]. Therefore, this paper uses the MPC approach and implements an adaptive prediction model. This allows the model to predict the next input to achieve the desired output based on all previous inputs and outputs, taking into account unpredictable disturbances or inherent batch variability in bioprocesses by updating the model parameters in real-time. The accuracy of the model fit when using the same model over different medium replacement conditions or different donors can even be below 50%. This is represented in Table A1 where the model for donor 2 is fitted on data of donor 3. This points out the variability between donors and realisations. However, the potential of the approach developed in this work is that the model is estimated and adapted in real-time solely using data for that specific realisation/individual, and thus guarantees a personalised approach.

This work also uses the concept of a soft sensor by using another measurable variable (lactate concentration) to estimate a desired critical quality attribute of the bioprocess (cell number). The flexibility of the controller to react to disturbances as well as process variability is shown by successfully applying the controller to three different donors and six different control strategies in triplicate.

The next step for this work is to implement the controller in real-time to the system and re-evaluate the performance of the controller. The prediction model will be updated with every new data point received from the current experimental run. In future experiments, the idea is to start from the known model structure, which was found to be the best representation for that bioprocess. In this case, the model would be a DARX model with a fixed $a_{1}$ parameter and a variable $b_{0}$ over time. After gathering enough data points, depending on the measuring frequency, this could be one day. The model will be developed based on the parameters defined by the process at hand. After this initial data gathering period, the model and controller will be updated in real-time using only the data from the current experiment. Using only the fixed model structure from previous experiments would lead to better results compared to other modelling techniques, where the parameter values of previous experiments are also used without tuning them based on experiment-specific data. The MPC approach presented in this work, which uses the data of each specific realisation, results in a model that adapts well to the process at hand.

In addition, this MPC model could potentially address a case study based on giving the process just enough medium to reach a certain percentage of the maximum cell number at harvest. This maximum cell number is estimated when supplying the process with 100% medium every day. However, to practically perform such a controlled process, additional knowledge about the system is required, which can be gained by performing follow-up experiments. One strategy to consider for these experiments is to observe the $b_{0}$ values of the DARX model, which is continuously re-estimated with every new data point collected from the experiment at hand. Further analysis could lead to finding certain thresholds for this parameter that would result in reaching a predefined percentage of the maximum achievable cell number at harvest.

Another additional path to explore is to correlate the cumulative lactate produced back to the biomass growth, in order to use the measurements as a soft sensor and estimate the amount of biomass at each lactate sample time point. However, the relation between the number of cells and lactate produced can differ not only between cell types, but also between different medium replacement strategies. In cases where cells receive a very low amount of medium, cells could die due to nutrient and growth factor depletion. Another possibility which could lead to a change in the relation between the amount of cells and the amount of lactate produced is a metabolic alteration (a by-product of glycolysis) by the cells when the amount of replaced medium is low [41]. Therefore, it would also be important in additional experiments to assess different quality attributes of the cells to check whether all process parameters are possible or if certain thresholds on medium replacement are required to avoid changing the quality and characteristics of the cells. The quality could be assessed with live/dead analysis, additional measures such as Lactate Dehydrogenase (LDH) and flow cytometry for MSC markers or the trilineage potential, determining the osteogenic, chondrogenic or adipogenic potential.

When these two steps of real time implementation and translation into cell numbers are combined, the controller could potentially solve case studies using an adaptive reference trajectory where a specific number of cells is required by a specific realistic time period using a minimal amount of medium. This approach is also capable of implementing different manipulated and controlled variables, in case new sensor techniques come onto the market.

Finally, instead of using well plates as a way to keep process costs low and experimental time short for a large amount of experiments, we envisage the use of such tools for suspension bioreactors where progenitor cell populations can be scaled-up for clinical production, allowing, at the same time, the capacity for real-time process adaptation [10,40].

## 5. Conclusions

The model predictive controller developed in this work is a generic algorithm which requires minimal effort to implement different process parameters and different responses of the system. This controller has the potential to be an inexpensive tool to minimise the costs and time of cell expansions in combination with assured product quality by design (QbD) [42]. Using cumulative lactate concentrations as an output measurement of the controller has proven to be useful in this specific bioprocess setting, where high glucose DMEM was used. However, it is important, when applying this method to a different bioprocess, to first assess which output measurement and related process parameter would suit that specific bioprocess.

Six different combinations of medium replacement were tested on three different donors in triplicate in order to model the dynamical response of medium replacement on cell proliferation. This dynamic response is best modelled using a DARX prediction model, resulting in an overall high R^2^ of 99.80% ± 0.02% for the DARX model on the same experimental data. The process–model mismatch is also low when applying a model based on experimental data from one triplicate to experimental data from another one of the triplicates. The average fit for the triplicates in DARX models on all the triplicates of experimental data is 96.57% ± 3.26%.

Based on simulations, the model predictive controller designed in this work shows promising results to accurately predict the effect of medium replacement on cell growth. The medium change input suggested by the simulation has a 86.45% ± 0.78% accuracy compared to the real experimental data, whereas the accumulated lactate output has an accuracy of 98.64% ± 0.10% compared to the target experimental data.

The results in this work show that this lactate-based model predictive controller can be applied to different donors as well as different medium-replacement strategies. The parameters are estimated for each individual experimental run, resulting in a high accuracy fit between the simulated data and the experimental data. Using these individualised parameters is the main advantage compared to other control strategies, which are more focused on a suitable prediction for the average bioprocess [14,17].

## Figures and Tables

**Table 1:** Overview of medium replacement strategies. The amount of medium replaced is indicated as a percentage of the total working volume of the well, which changed over the different days.

Medium Replaced		Day				
		1	2	3	4	Explanation
**Condition**	**1**	10.0	10.0	25.0	45.0	Increasing: 0; 15; 20
	**2**	12.5	25.0	36.5	50.0	A steady increase of 12.5
	**3**	10.0	20.0	35.0	45.0	A decreasing increase: 10; 15; 10
	**4**	10.0	10.0	10.0	10.0	Constant replacement
	**5**	50.0	50.0	50.0	50.0	Constant replacement
	**6**	100.0	100.0	100.0	100.0	Constant replacement

**Table 2:** Total amount of cells harvested after the cell culture expansion, which is averaged over the triplicates for each donor and condition (100,000 cells).

Harvested Cells		Donor		
		1	2	3
Condition	1	1.48 ± 0.13	0.88 ± 0.22	1.31 ± 0.18
	2	1.69 ± 0.11	1.63 ± 0.13	1.75 ± 0.12
	3	1.48 ± 0.10	1.45 ± 0.33	1.73 ± 0.24
	4	0.96 ± 0.25	0.65 ± 0.04	1.10 ± 0.38
	5	1.99 ± 0.19	1.61 ± 0.18	1.89 ± 0.26
	6	2.37 ± 0.21	2.24 ± 0.25	2.08 ± 0.22

**Table 3:** Efficiency of medium used over the total cell culture period, calculated by dividing the total cell numbers by the total amount of medium used (10,000 cells mL^−1^).

Medium Efficiency		Donor		
		1	2	3
**Condition**	**1**	7.79 ± 0.71	4.61 ± 1.15	6.91 ± 0.93
	**2**	7.53 ± 0.48	7.29 ± 0.60	7.79 ± 0.55
	**3**	7.06 ± 0.45	6.88 ± 1.55	8.21 ± 1.15
	**4**	6.85 ± 1.79	4.67 ± 0.29	7.86 ± 2.70
	**5**	6.64 ± 0.63	5.38 ± 0.61	6.29 ± 0.88
	**6**	4.73 ± 0.41	4.48 ± 0.51	4.17 ± 0.44

**Table 4:** The accumulated lactate divided by the cell numbers at the end of the cell culture period and divided by total culture time (120 h), averaged over the triplicates for each donor and condition (10,000,000 mM cell^−1^ h^−1^).

Lactate Production		Donor		
		1	2	3
**Condition**	**1**	6.71 ± 0.65	11.49 ± 2.84	10.07 ± 1.55
	**2**	6.16 ± 0.33	6.29 ± 0.65	7.83 ± 0.67
	**3**	7.27 ± 0.66	7.26 ± 1.65	8.18 ± 1.38
	**4**	11.78 ± 3.93	13.35 ± 0.88	12.09 ± 4.21
	**5**	6.14 ± 0.99	7.09 ± 0.89	7.06 ± 1.11
	**6**	5.27 ± 0.40	5.40 ± 0.69	6.44 ± 0.65

**Table 5:** Accuracy measured using normalised root mean square error (NRMSE) of DARX model compared to output of the interpolated data for each condition, donor and triplicates. The DARX model used is either the one based on the corresponding experimental data (diagonal values) or on the data of an experimental triplicate. The value is NRMSE multiplied by 100 and expressed as a percentage, with 100 being a perfect fit.

NRMSE			Donor 1			Donor 2			Donor 3		
			DARX Model Triplicate								
Condition			1	2	3	1	2	3	1	2	3
**1**	**Experimental data triplicate**	**1**	99.76	96.17	94.72	99.77	97.11	95.63	99.79	93.83	98.15
		**2**	96.28	99.77	91.74	97.17	99.78	93.34	94.08	99.79	92.77
		**3**	94.65	91.42	99.76	95.50	93.00	99.78	98.12	92.45	99.79
**2**		**1**	99.78	93.27	97.14	99.80	95.59	98.14	99.82	91.97	94.21
		**2**	93.56	99.79	95.87	95.73	99.80	94.17	92.42	99.80	97.16
		**3**	97.21	95.78	99.79	98.06	93.94	99.78	94.47	97.13	99.80
**3**		**1**	99.79	93.34	98.64	99.80	98.30	95.47	99.82	98.02	93.74
		**2**	93.66	99.79	92.78	98.29	99.80	95.19	97.99	99.82	94.68
		**3**	98.67	92.39	99.80	95.34	95.02	99.79	93.49	94.55	99.81
**4**		**1**	99.75	99.13	92.60	99.75	97.45	95.98	99.77	90.54	95.41
		**2**	99.22	99.77	93.26	97.46	99.74	93.62	91.10	99.77	95.41
		**3**	92.20	92.93	99.77	95.87	93.31	99.76	95.55	95.26	99.77
**5**		**1**	99.81	98.30	87.19	99.82	98.54	95.71	99.83	94.76	91.61
		**2**	98.31	99.81	86.28	98.54	99.82	96.61	94.58	99.81	96.52
		**3**	85.80	84.63	99.82	95.58	96.52	99.82	91.12	96.43	99.81
**6**		**1**	99.80	94.34	94.67	99.83	96.96	98.84	99.82	97.13	94.34
		**2**	94.53	99.81	90.32	97.03	99.84	97.43	97.08	99.82	96.84
		**3**	94.44	89.54	99.82	98.90	97.39	99.84	94.10	96.75	99.81

**Table 6:** The accuracy of the MPC simulation is measured using NRMSE and multiplied by 100 to be expressed as a percentage, with 100 being a perfect fit. The accuracy of the MPC simulation is represented for the difference in input (accumulated replaced medium (mL)) of the experimental data compared to the input of the simulated data. All NRMSE values calculated for all three donors and all three triplicates are equal for the same condition of medium replacement.

Condition	1	2	3	4	5	6
**NRMSE**	84.96	86.28	86.18	87.18	87.07	87.03

**Table 7:** The accuracy of the MPC simulation is measured using NRMSE and multiplied by 100 to be expressed as a percentage, with 100 being a perfect fit. The accuracy of the MPC simulation is represented as the difference between output (accumulated lactate (mM)) of the experimental data compared to the output of the simulated data. The DARX model used to perform the simulation is either the one based on the corresponding experimental data (diagonal values) or on the data of an experimental triplicate.

NRMSE			Donor 1			Donor 2			Donor 3		
			DARX Model Triplicate								
Condition			1	2	3	1	2	3	1	2	3
**1**	**Experimental data triplicate**	**1**	98.76	98.77	98.77	98.73	98.72	98.73	98.65	98.64	98.65
		**2**	98.76	98.76	98.77	98.73	98.73	98.73	98.64	98.63	98.64
		**3**	98.82	98.82	98.83	98.68	98.67	98.68	98.67	98.66	98.67
**2**		**1**	98.70	98.70	98.71	98.67	98.66	98.66	98.58	98.57	98.59
		**2**	98.70	98.70	98.71	98.68	98.67	98.67	98.57	98.56	98.58
		**3**	98.71	98.71	98.72	98.67	98.66	98.66	98.59	98.58	98.60
**3**		**1**	98.67	98.67	98.68	98.63	98.62	98.63	98.58	98.56	98.61
		**2**	98.68	98.68	98.69	98.65	98.64	98.65	98.56	98.54	98.59
		**3**	98.68	98.68	98.69	98.61	98.61	98.62	98.56	98.54	98.59
**4**		**1**	98.77	98.78	98.78	98.83	98.82	98.84	98.67	98.66	98.67
		**2**	98.76	98.78	98.78	98.85	98.84	98.85	98.67	98.65	98.66
		**3**	98.77	98.78	98.78	98.82	98.81	98.82	98.67	98.66	98.67
**5**		**1**	98.60	98.59	98.60	98.64	98.64	98.64	98.51	98.50	98.51
		**2**	98.62	98.62	98.63	98.65	98.65	98.65	98.53	98.52	98.53
		**3**	98.56	98.56	98.57	98.64	98.64	98.64	98.51	98.50	98.51
**6**		**1**	98.55	98.55	98.56	98.53	98.53	98.53	98.47	98.47	98.47
		**2**	98.53	98.53	98.54	98.53	98.53	98.54	98.48	98.48	98.48
		**3**	98.49	98.50	98.50	98.51	98.51	98.51	98.47	98.47	98.47

## Appendix A

**Table A1:** Model mismatch example, where the DARX models of donor 2 are applied to experimental data from donor 3. The results are the NRMSE multiplied by 100, between the DARX model and the data.

NRMSE			DARX Model Donor 2 Triplicates		
Condition			1	2	3
**1**	**Experimental data triplicate donor 3**	**1**	54.16	50.70	59.50
		**2**	61.82	58.50	66.99
		**3**	52.98	49.47	58.27
**2**		**1**	56.75	50.97	58.41
		**2**	66.54	61.10	68.14
		**3**	63.83	58.28	65.50
**3**		**1**	57.66	56.96	63.13
		**2**	55.66	54.89	61.14
		**3**	49.32	48.60	55.05
**4**		**1**	51.67	48.30	57.18
		**2**	63.02	59.82	68.15
		**3**	57.41	54.13	62.73
**5**		**1**	87.32	87.72	90.86
		**2**	82.54	83.28	86.94
		**3**	78.60	79.38	83.17
**6**		**1**	90.43	87.27	89.94
		**2**	87.54	84.30	86.98
		**3**	84.36	81.07	83.82
